# Does Seeing What Others Do Through Social Media Influence Vaccine Uptake and Help in the Herd Immunity Through Vaccination? A Cross-Sectional Analysis

**DOI:** 10.3389/fpubh.2021.715931

**Published:** 2021-11-02

**Authors:** Abrar Al-Hasan, Jiban Khuntia, Dobin Yim

**Affiliations:** ^1^College of Business Administration, Kuwait University, AlShadadiyah City, Kuwait; ^2^CU Denver Business School, Information Systems Department, University of Colorado, Denver, CO, United States; ^3^Sellinger School of Business, Information Systems Law and Operations Department, Loyola University Maryland, Baltimore, MD, United States

**Keywords:** social media, COVID-19, vaccine, health belief model (HBM), herd immunity

## Abstract

Widespread acceptance of COVID-19 vaccination is the next major step in fighting the pandemic. However, significant variations are observed in the willingness to take the vaccination by citizens across different countries. Arguably, differences in vaccination intentions will be influenced by beliefs around vaccines to influence health. Often perceptions of what others are doing and the information available guide individuals' behaviors for vaccination. This is more so in the digital age with the influence of the internet and media. This study aims to determine the factors that impact willingness to vaccinate for COVID-19. We examined factors associated with acceptance of vaccine based on (1) constructs of the Health Belief Model (HBM), (2) sources of information, (3) social media usage, (4) knowledge of COVID-19 treatment, and (5) perception of government's efforts for mitigation. Randomly sampled online survey data was collected by a global firm between December 2020 and January 2021 from 372 citizens (with a response rate of 96.6%) from multiple regions, including North America, the Middle East, Europe, and Asia. Ordered probit regression suggests that the health belief model constructs hold. Perceived severity of COVID-19 (*P* < 0.001) and action cues of others taking the vaccine positively influences a subject's vaccine intent (*P* < 0.001), perceived benefits and perceived efficacy of the vaccine positively influences a subject's vaccine intent (*P* < 0.001). Perceived barriers negatively influence vaccine intent (*P* < 0.001). Interestingly as for media usage, mainstream media (e.g., TV, newspaper) (*P* = 0.006) and social media (*P* = 0.013) both negatively influence a subject's vaccine intent. Social media platforms that are more entertainment and social-based, such as Whatsapp, Instagram, and YouTube, have a negative and significant influence on vaccine intent (*P* = 0.061), compared to other more information-based social media platforms (e.g., Twitter, LinkedIn). Knowledge of COVID-19 treatment positively influences vaccine intent (*P* = 0.023). Lastly, governmental efforts' perceived reliability in mitigation strategy (*P* = 0.028) and response efforts (*P* = 0.004) negatively influence vaccine intent. The study highlights the “wait-and-see” action cue from others and leaders in the community. It also informs the importance of shaping media information for vaccination through informative media and social media outlets to counteract any misinformation.

## Introduction

Experts suggest that herd immunity to achieve a 60–90% threshold is a way to end the COVID-19 pandemic (1). A population reaches herd immunity when the average number of people infected by a single sick person falls below one. A patient may infect another, but that second patient cannot infect a third. A vaccine helps individuals' immune systems to develop protection from disease. A vaccine is not a cure by itself. Vaccination, through a “process of administration and distribution, reaching to a sufficiently large percentage of a population, provides herd immunity,” that in turn, protects the vaccinated as well as the non-vaccinated who may be immune-compromised and cannot get a vaccine because even a weakened version would harm them (2, 3). A plethora of research suggests that vaccination is the most effective method of preventing several infectious diseases, such as smallpox, polio, and tetanus (4–7). Relatedly, low vaccination rates have kept the measles outbreak still a concern (8, 9). The countdown to reach herd immunity in the case of COVID-19 has started with the vaccination. Several vaccines are being distributed, and most of them are highly effective (10). However, there is a concern that the vaccine's uptake against the transmission and mutation of the virus may not be enough to manage herd immunity (11–13).

Concerns arise as to whether the speed at which people are getting vaccinated is fast enough to balance the speed at which the virus is still spreading through circulation, and the evolving new strains at which reaching a threshold of herd immunity may not be possible. The situation is even more aggravating when more than 15% of Americans oppose taking the vaccine (14). To substantially reduce morbidity and mortality from COVID-19, an efficacious and safe vaccine must be delivered swiftly and broadly to the public as soon as it is available (15). Denial or refusal to be vaccinated, partial vaccination, or have only one family member to be vaccinated is coined as “vaccine hesitancy,” which has emerged as a contradiction to the overwhelming scientific agreement on safety and efficacy of vaccines (16–18). Such hesitancy stems from mostly individuals' mistrust, lack of confidence, complacency, not seeing the value, access, convenience, and many other factors, but compounded through the ethical debates to make vaccination legal in countries or states (19). Vaccine hesitancy is a significant barrier to vaccine uptake and the achievement of herd immunity, which is required to protect the most vulnerable populations (15).

While vaccine hesitancy has been examined in previous literature on several viruses, the proliferation of anti-vaccination misinformation through social media and various available sources of information in the context of the COVID-19 pandemic has given this stream of literature new urgency (20, 21). The multifaceted nature of willingness to accept the COVID-19 vaccine entails different factors. For instance, vaccine hesitancy maybe because of informational, psychologic, socio-demographic, and cultural factors (22–25). Prior research points greatly toward the socio-economic determinants of vaccine hesitancy, involving qualitative single-country work (26–28) to large scale surveys across dozens of countries (20, 29, 30). Similarly, studies have also discussed the psychological determinants of vaccine hesitancy (22, 31). Some studies have pointed to the information aspects of vaccine hesitancy, i.e., the role of availability of information (and misinformation) on vaccination intention (21, 26–28, 32–36). During the COVID-19 pandemic, information has been abundant. Media channels covered the latest developments (37). Both traditional and social information channels provided a lot of information. Prior research suggests these information avenues have a significant role in promoting either vaccination willingness or vaccine hesitancy (21, 38–41). The COVID-19 vaccine rollout is ongoing. However, reports suggest that refusal for vaccination is also high (20, 42, 43). Thus, it is vital to further investigate the role of information sources on COVID-19 vaccine rollout. This study, motivated by this concurrent challenge, extends the research around the role of information sources on the intention to vaccinate. This study seeks questions on the proposed effect of informational impact in terms of sources, knowledge, and social media and its various types on vaccination intention, and includes other related information factors that have been shown to impact vaccine hesitancy such as psychological impact of health information and perception of governmental efforts.

This study asks the research question: *Why are some citizens not willing to vaccinate? What reasons can be drawn from the earlier research of similar vaccination contexts that can inform policymakers to take some actions to convince citizens for vaccinations?* Significant variations are observed in the willingness to vaccinate by citizens across different countries (44–48); appropriate tools and mitigation techniques are sparse in the hands of policymakers. These challenges, along with the beliefs around vaccines influencing health, aggravate the third COVID-19 spread around the Delta variant in several countries. Thus, this study's research question assesses the informational factors that impact willingness to vaccinate for COVID-19. While a myriad of reasons may exist why some citizens are not willing to vaccinate, this study is guided by a stream of research relevant to anchoring to the informational aspects of COVID-19 and citizens' perceptions of the vaccine. As highlighted next, we focus on five major areas derived from extant literature: (1) constructs of the Health Belief Model (HBM), (2) sources of information, (3) social media usage, (4) knowledge of COVID-19 treatment, and (5) a reliable perception of government's efforts for mitigation. We elaborate on these reasons next.

First, the information on the severity of the disease may be undermined. There may be a perception that COVID-19 is a temporary phenomenon and will decline automatically. In this context, people may believe that vaccine is not a need (49). As such, there have been several “conspiracy theories” floating around on the internet and elsewhere about COVID-19 (50). These unvalidated rumors put less emphasis on the severity of COVID-19 as a disease (51, 52). The linkage between severity and action regarding health is well-established in the existing health-belief-model-related prior research (53–55), thus using this model in this study to explore several belief-oriented factors associated with the COVID-19 vaccination uptake. Recent studies have examined the health-belief model to predict vaccine willingness in Hong Kong (56) and Malaysia (53). However, empirical examination of the severity-vaccine willingness in the COVID-19 context is sparse yet essential.

Second, there may be a genuine fear that vaccination has several unintended consequences, apart from side effects (57). Subsequently, a “wait and watch” principle may be adopted by citizens. Such a fearful perception may inhibit their ability to embrace the vaccine. However, as postulated by the prior health belief model relevant work, others' adoption of health practices may send actionable cues to a focal person to adopt the practice (53, 58–60). That being the case in vaccination, a significant population may wait for how it affects others and then decide whether to take the vaccine (61). Thus, exploring whether the willingness to vaccinate is higher or lower by seeing what others are doing in their social media is a salient differentiator to inform vaccine uptake.

Third, people worldwide are increasingly consulting the internet, social media, and their social networks for health information, enhancing the role of media in public health promotion (62–65). Thus, whether information passed through media can influence vaccine intent remains a concern (66). However, there is also substantial potential for harmful misinformation to spread across media platforms fueling vaccine hesitancy (67). Media worldwide on COVID-19 has been instilled with a lot of misinformation (68–70). A recent analysis of the most viewed coronavirus YouTube videos found that over 25% of the top videos contained misleading information reaching millions of viewers worldwide, terming the pandemic a pandemic of misinformation (71). Repeated visibility of misinformation through media outlets daily causes repeated exposure known to increase fake news beliefs (13, 72). Different media outlets (e.g., social media, television, newspapers, etc.) have differing characteristics, thus the interpretations of COVID-19 vaccination information may also differ. Therefore, it is essential to understand better different media sources' influence on vaccine willingness (73).

Fourth, the question as to whether individuals with higher knowledge of COVID-19 are more willing to vaccinate remains unexplored (68). The gap in knowledge, especially with the mass amount of misinformation in media worldwide, may contribute to the increased risk of infection. Positive attitude and behavioral changes are driven by knowledge and perceptions toward preventive practices (74). Examining whether and what type of knowledge enacts COVID-19 vaccination is essential to ending this pandemic.

Finally, governments worldwide and health organizations have not been able to mitigate and manage the pandemic well-enough. Research has shown that citizens have lost trust in health organizations because some were politicized during the pandemic (75). Throughout the pandemic, there has been low trust in governments, as manifested through the spread of misinformation, defiance of public health guidelines, and vaccine hesitancy (76). However, to reach herd immunity, rebuilding government legitimacy is essential. One way of enhancing trust is through transparency and disclosure of accurate information (77). Decisive government response to the virus has also shown an increase in trust (78). Therefore, examining citizens' perception of mitigation efforts of the government influences vaccine intent is essential to reach herd immunity.

To summarize, following through with HBM, we posit that vaccine intent will be influenced by what others are doing and the information available to guide their behaviors. This influence is also displayed more so in the digital age in the media's mass influence. Knowledge of COVID-19 treatment and reliable perception of government efforts for mitigation all may influence vaccination behavior. While the vaccine's benefits are clear (79), citizens need to be affirmed and confirmed to take the vaccine. The limitations lie in convincing them well-enough with the right knowledge and possibly, utilizing media outlets appropriately. This study addresses these issues *via* a perceptual survey of citizens across varying societies.

## Methods

### Recruitment

This study started with a discussion in a focus group of ten people. The focus group participants opined that assessing the HBM and informational aspects (sources of information, social media, knowledge, and perception of governmental efforts) is essential to manage vaccine intent. The focus group also suggested that different lifestyles and mindsets will differ in vaccine intent. This suggestion motivated this study to explore the research question across different countries with polarized mindsets and different lifestyles.

A global survey-deploying firm collected the data for this study using various online media outlets and organizations to spread the survey. The firm recruited respondents from North America, Middle East, Europe, and Asia between December 2020 to January 2021. The firm sampled respondents using age, gender, ethnicity, and geographic region-based strata and quota matching process by sampling a proportional number of individuals relative to the specified population. Participation in the survey was free and voluntary—the respondents filled in electronic informed consent that was shown on the first page of the survey (i.e., written consent). The firm protects the confidentiality of anonymous respondents. The data were analyzed and received anonymously. There were no minors included in the study.

Data was collected using a survey instrument. The questions asked participants about their opinion on the COVID-19 vaccination. The survey items included the willingness to vaccinate questions, knowledge of COVID-19 questions, information-seeking questions, social media usage, government perception, along with health belief model constructs and several existing validated scales from prior studies (80, 81). The survey instrument was pilot tested using a sample of 18 respondents, leading to minor refinements to a few items. A total of 385 participants took the survey. Because of missing responses to the items, 13 observations were excluded, resulting in a sample size of 372. Responses were coded, validated, and analyzed using STATA version 16 (StataCorp).

### Sample Demographics

Table 1 describes the variables used in this study. We present the coding scheme of the variables from the survey in Appendix Table 1. Table 2 shows the descriptive statistics and pairwise correlations amongst the key variables used in this study. Appendix Table 2 shows the demographic descriptive statistics, and as displayed 224 out of 378 (59.9%) of the sample were female. In terms of age group, the 18–27 age group make 33.1% of the sample (125 out of 378), the 28–37 age group make 38.4% of the sample (145 out of 378), the 38–47 age group make 16.9% of the sample (64 out of 378), the 48–57 age group make 5.6% of the sample (21 out of 378), and the 58 and over age group make 6.1% of the sample (23 out of 378).

**Table 1:** Description of variables in this study.

**Variable**	**Description**
INTENT	Individual's intention to take COVID-19 vaccine.
ACTION CUES (ACT. CUE.)	This is the stimulus needed to trigger the decision-making process to take the COVID-19 vaccine. Triggers for receiving COVID-19 vaccination by 4 items, including if others in the community take it, if the leader of the country takes it, if the leader of our community takes it, if the doctors recommend it.
SEVERITY	Perceived severity or degree of harm from engaging in unhealthy behavior; the extent to which one will experience suffer or die from contracting COVID-19.
BENEFITS	This refers to a person's perception of the effectiveness of the COVID-19 vaccine to reduce the threat of the disease.
BARRIERS	This refers to a person's feelings on the obstacles to taking the COVID-19 vaccination.
EFFICACY	Perceived of the vaccine efficacy against COVID-19.
IS_TRADITIONAL MEDIA (IS_TRAMED.)	The extent to which an individual uses health information sources to attain COVID-19 information from traditional media, e.g., TV and newspaper.
IS_SPECIAL HEALTH (IS_SPHLTH.)	The extent to which an individual uses health information sources to attain COVID-19 information from health specialists, e.g., Doctors and health websites.
IS_SOCIAL MEDIA (IS_SM)	The extent to which an individual uses health information sources to attain COVID-19 information from social media.
SM_SHORTVIDEO (SM_S.VID.)	The extent to which an individual uses short video social media platforms (e.g., Snapchat, TickTock) to attain COVID-19 information.
SM_COMMUNITY (SM_COM.)	The extent to which an individual uses community-based social media platforms (e.g., Facebook, LinkedIn) to attain COVID-19 information.
SM_INFORMATION (SM_INF.)	The extent to which an individual uses informational social media platforms (e.g., Twitter) to attain COVID-19 information.
SM_ENTERTAINMENT (SM_ENT.)	The extent to which an individual uses entertainment social media platforms (e.g., Whatsapp, Instagram, and YouTube) to attain COVID-19 information.
GOV_STRATEGY (GOV_STR.)	Perceived effectiveness of government mitigation strategy against COVID-19.
GOV_PERFORMANCE (GOV_PERF.)	Perceived effectiveness of government performance against COVID-19 policy.
KNOW_SYMPTOMS (KN._ SYM.)	The extent to which one is aware or knowledgeable about COVID-19 symptoms.
KNOW_TREATMENT (KN._TRT.)	The extent to which one is aware or knowledgeable about COVID-19 and relevant situations.
AVAILABILITY	The extent of the availability of the COVID-19 vaccination in the citizens' country.
COUNTRY	Country of residence.
AGE	Age of respondent.
GENDER	Gender of the respondent.
INCOME	Household income of the respondent.
ETHNICITY	Ethnicity of the respondent.

**Table 2:** Summary statistics and pairwise correlations amongst key variables (*N* = 372).

	**Variables**	**Mean (SD^a^)**	**Min**	**Max**	**1**	**2**	**3**	**4**	**5**	**6**	**7**	**8**	**9**	**10**	**11**	**12**	**13**	**14**	**15**
1	INTENT	3.92 (1.24)	1	5	1.00														
2	ACT. CUE.	0 (0.64)	−1	1	0.27	1.00													
3	SEVERITY	0 (0.80)	−2	1	0.25	0.07	1.00												
4	BENEFITS	0 (0.90)	−3	1	0.55	0.23	0.09	1.00											
5	BARRIERS	0 (0.79)	−1	1	−0.67	−0.08	−0.02	−0.43	1.00										
6	IS_TRAMED.	−0.01 (0.73)	−1	2	−0.06	0.02	0.06	−0.01	0.06	1.00									
7	IS_SPHLTH.	0.00 (0.44)	−1	1	0.09	0.08	0.07	0.12	−0.09	0.07	1.00								
8	IS_SM	−0.01 (1)	−1	1	−0.16	0.19	0.01	−0.06	0.18	0.03	−0.03	1.00							
9	SM_S.VID.	0.01 (1)	−1	3	−0.06	0.08	0.05	−0.02	0.11	0.13	0.04	0.15	1.00						
10	SM_COM.	0.01 (1.01)	−1	3	0.03	0.05	0.11	0.05	0.00	0.15	−0.02	0.11	0.00	1.00					
11	SM_INF.	−0.01 (1)	−1	1	−0.01	0.21	0.01	0.05	0.05	−0.21	0.01	0.34	0.00	0.00	1.00				
12	SM_ENT.	−0.01 (0.70)	−1	2	−0.19	0.10	−0.06	−0.06	0.17	0.20	0.03	0.27	0.07	0.02	0.01	1.00			
13	GOV_STR.	1.20 (0.48)	1	3	−0.17	−0.11	−0.12	−0.14	0.09	0.02	−0.03	0.04	−0.04	0.05	−0.07	0.02	1.00		
14	GOV_PERF.	3.14 (1.17)	1	5	−0.22	0.09	−0.19	−0.12	0.23	−0.10	0.01	0.09	0.17	−0.23	0.16	0.22	−0.11	1.00	
15	KN._ SYM.	4.85 (1.24)	1	7	0.04	−0.11	−0.02	0.07	−0.08	0.06	0.05	−0.06	−0.03	0.14	−0.17	0.03	0.02	−0.19	1.00
16	KN._TRT.	1.59 (1)	−2	4	0.16	0.12	0.03	0.08	−0.05	0.07	0.09	0.04	−0.04	−0.02	0.08	−0.03	−0.11	−0.10	0.10

a *SD, Standard deviation*.

As for household income level, 81 out of 378 (21.4%) of the sample make < $30,000 annually, 54 out of 378 (14.3%) make $30,000–50,000, 57 out of 378 (15.1%) make $50,000–80,000, 47 out of 378 (12.4%) make $80,000–100,000, 50 out of 378 (13.2%) make $100,000–150,000, and 89 out of 378 (23.5%) make more than $150,000.

In terms of ethnicity, 244 out of 376 (64.9%) of the sample described themselves as Middle Eastern, 84 out of 376 (22.3%) as White or Caucasian, 30 out of 376 (8.0%) as Black or Latin, and 18 out of 376 (4.8%) as Asian. We note that the sample is not representative of any particular ethnicity, but a diverse mix of ethnicities.

For the place of residence, 238 out of 378 (63.0%) of the sample live in the Middle East, 111 out of 378 (29.4%) live in North America, 15 out of 378 (4.0%) live in Europe, and 14 out of 378 (3.7%) live in Asia. We note that Europe and Asia have several countries with different contexts and policies, differing COVID-19 infections, vaccination plans; and thus, the sampling of countries does not reflect any specific policy representation. A detailed distribution of several demographic controls used in the models is available in Appendix Table 2.

### Study Variables

**Figure 1:**
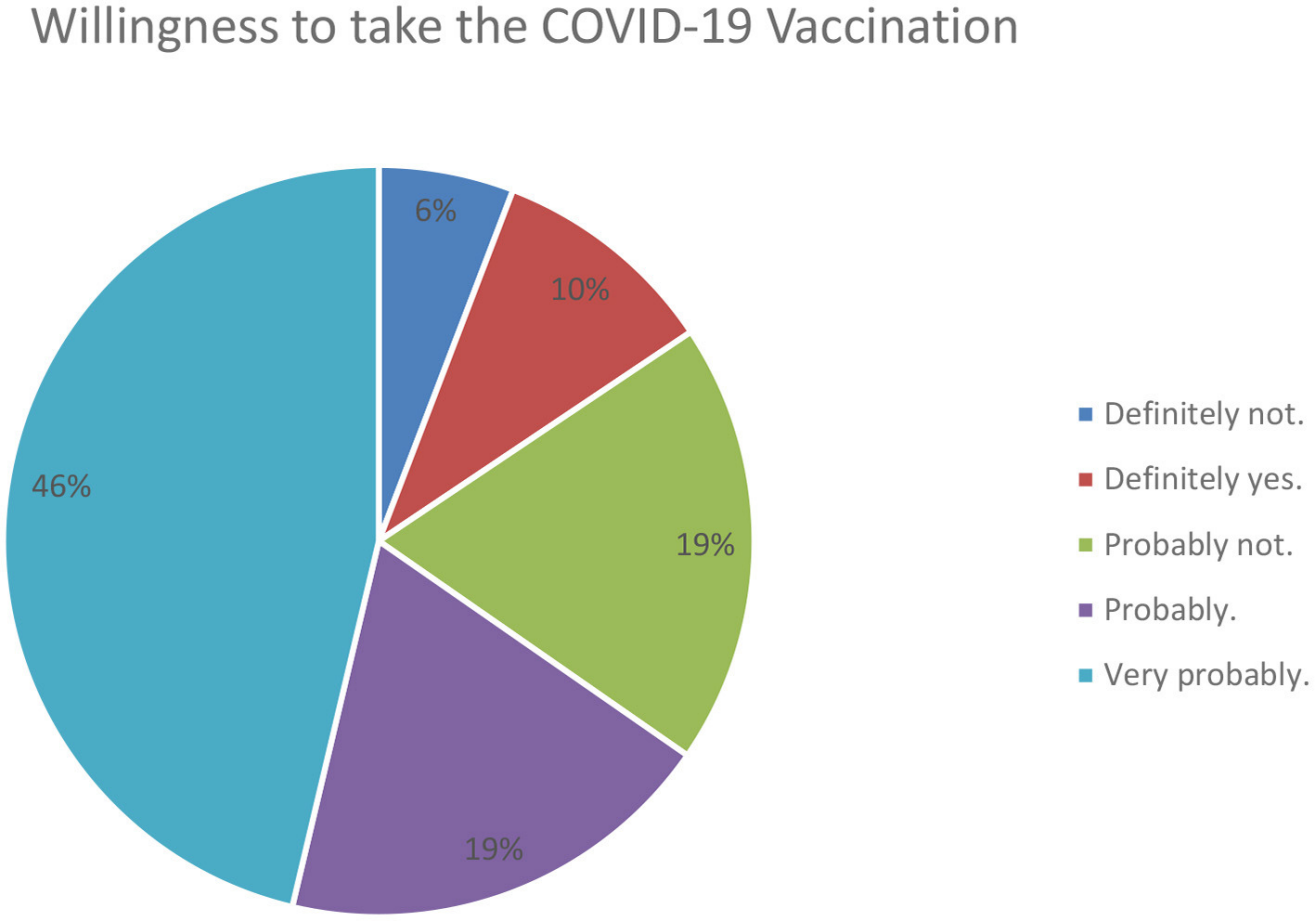
COVID-19 willingness to vaccinate *N* = 372.

The primary dependent variable in this study is INTENT to vaccinate for COVID-19. As shown in Appendix Table 1, INTENT is measured on a Likert scale of 1 to 5 whether they intended to take the vaccine. Table 2 displays that, on average, INTENT is 3.92 out of 5, showing a high willingness to take the vaccine if offered. Figure 1 displays the INTENT for the sample, showing that on average, 10% of the sample would definitely vaccinate, 46% would very probably vaccinate, 19% would probably vaccinate, 19% would probably not vaccinate, and 6% would definitely not vaccinate.

Five sets of independent variables were of interest in this study: first, the HBM constructs; second, the sources of COVID-19 information; third, the usage of social media; fourth, the knowledge of COVID-19; and fifth, the perception of the governmental efforts.

HBM constructs compose of action cues, severity, efficacy, benefits, and barriers to vaccinate for COVID-19. These variables were operationalized using previous scales (see Appendix Table 1), and multi-items for reflective constructs were tested for interitem correlation and Cronbach's alpha. The constructs were standardized, and a single variable was generated using a new scale. Further details are provided in Appendix Table 3.

ACTION CUES is defined as triggers for receiving COVID-19 vaccination that include if others in the community take it, if the leader of the country takes it, if the leader of the community takes it, and if the doctors recommend it. The items' internal consistency was tested using Cronbach α (0.64), and the standardized score was generated for the response variable. As Table 2 shows, the variable response has a mean of 0.00 and a standard deviation of 0.64. SEVERITY is defined as perceived severity or degree of harm from engaging in unhealthy behavior; the extent to which one will experience suffer or die from contracting COVID-19. The items' internal consistency was tested using Cronbach α (0.71), and the standardized score was generated for the response variable. The variable has a mean of 0.00 and a standard deviation of 0.80. The perceived BENEFITS of the vaccination has a mean of 0.00 and a standard deviation of 0.90. The BARRIERS measures the obstacles to receive COVID-19 vaccination, such as concerns about the safety and possible side effects of the COVID-19 vaccination. The items' internal consistency was tested using Cronbach α (0.80), and the standardized score was generated for the response variable. The BARRIERS have a mean of 0.00 and a standard deviation of 0.79.

For the health information sources of COVID-19, and social media platform use for COVID-19 information variables, each categorical response from the multi-category items were transformed into dummy variables. Factor analyses using principal factor for estimation with no rotation were performed to identify factors latent to the information search constructs. We have provided detailed analyses in the Appendix Tables 4–7.

The respondents were asked where they obtained health information on COVID-19, whether from social media, TV, newspapers, or doctors and health care specialists. Information Source (IS) was measured using a count of the total number of information sources used to attain COVID-19 information. Factor analysis led us to three main types of sources were measures (1) traditional media sources such as TV and newspapers, (2) specialized health sources such as doctors and health websites, (3) and social media sources. Both IS_TRADITIONAL MEDIA and IS_SOCIAL MEDIA had a negative mean of −0.01, whereas IS_SPECIAL HEALTH had a positive mean of 0.00 with a standard deviation of 0.44.

Social media use examines the extent to which an individual was using specific social media platforms to attain COVID-19 information. Factor analysis allowed us to categorize social media platforms into four main categories: (1) Short video-based social media (e.g., Snapchat and TickTock), (2) Social communities (e.g., Facebook and LinkedIn), (3) Informational social media (e.g., Twitter), (4) and lastly social media for entertainment and social purposes (e.g., Whatsapp, Instagram, and YouTube). The mean for SM_INFORMATION and SM_ENTERTAINMENT was −0.01 with a standard deviation of 1.00 and 0.70, respectively. On the other hand, SM_SHORTVIDEO and SM_COMMUNITY were 0.01 with a standard deviation of 1.00 and 1.01, respectively.

The knowledge variable was coded to reflect the respondents' overall knowledge of COVID-19 using two types of knowledge: (1) KNOW_SYMPTOMS: knowledge about the symptoms of COVID-19, and (2) KNOW_TREATMENT: knowledge about the treatment of COVID-19 (see Appendix Table 1). The mean for KNOW_SYMPTOMS was 4.85 showing good knowledge of the symptoms of COVID-19. Yet the mean for KNOW_TRMT was 1.59 showing a lower level of knowledge on the treatment of COVID-19.

Lastly, governmental efforts were measured using two variables: (1) GOV_STRATEGY, which measures the perceived effectiveness of the governmental mitigation strategy thus far in the pandemic, and (2) GOV_PERFORMANCE measures the perceived effectiveness of the governmental performance against COVID-19 policy. While the perceived government mitigation strategy has a mean of 1.20, the governmental performance perceived effectiveness is 3.14, with standard deviations of 0.48 and 1.17.

In addition to these key variables of interest, several control variables, as mentioned in Appendix Table 1, are included to account for counterfactual explanations relevant to our models.

### Econometric Analysis

The empirical model specifies the willingness of individuals to vaccinate for COVID-19. The independent variables we focused on include HBM constructs (ACTION CUES, SEVERITY, BENEFITS, BARRIERS), information source for COVID-19 information, social media usage for COVID-19 information, knowledge of symptoms, and knowledge of treatment COVID-19, governmental efforts. A set of control variables to enhance our empirical model's robustness include demographic characteristics of the survey participants, such as gender, age group, household income, ethnicity, and vaccine availability. The empirical model is described below:


(1) $$\begin{array}{l} INTENT\;TO\;VACCINATE_{i}\;\\ \;=\;\beta_{0}\;+\;\beta_{\text{1}}\times ACTION\_CUES_{i}\;+\;\beta_{\text{2}}\times SEVERITY_{i}\;\\ \;+\;\beta_{\text{3}}\;\times BARRIERS_{i}\;+\;\beta_{\text{4}}\;\times BENEFITS_{i}\;\\ \;+\;\beta_{\text{5}}\;\times COVID-19\;INFORMATION\;SOURCES_{i}\;\\ \;+\;\beta_{\text{6}}\times \;COVID-19\;SOCIAL\;MEDIA_{i}\;\\ \;+\;\beta_{\text{7}}\;\times COVID-19\;KNOWLEDGE_{i\;}\;\\ \;+\;\beta_{\text{8}}\;\times GOVERNMENTAL\;EFFORTS_{i\;}+\;Controls_{i}\;+\;\varepsilon_{i} \end{array}$$

Since the dependent variables are ordinal values, we used an ordered probit model to estimate the key variables' parameters with robust standard errors. Last, ε are disturbances associated with each observation. Using ordered probit regression, we estimated to what extent our set of crucial variables influence willingness to vaccinate for COVID-19.

## Results

Table 3 presents the estimation results for Equation (1). Table 3 columns (1–2) show the regression model to estimate INTENT with social media as an information source. Table 3 columns (3–4) displays the regression model to estimate INTENT with social media broken down into the four identified categories. Furthermore, since BARRIERS and BENEFITS were highly correlated (−0.43), the two variables were not included in the same regression model to avoid multicollinearity issues. Table 3 column (1) and column (3) shows the parameter estimates for the INTENT dependent variable with BENEFITS in the model, and Table 3 column (2) and column (4) shows the parameter estimates for the INTENT dependent variable with BARRIERS in the model.

**Table 3:** Ordered probit regression models.

	**Social media as**		**Social media**	
	**info. source**		**platform types**	
	**(1)**	**(2)**	**(3)**	**(4)**
**Dependent variable: vaccine intention**				
ACTION CUES	0.434	0.484	0.409	0.476
	(*P* < 0.001)^a^	(*P* < 0.001)	(*P* < 0.001)	(*P* < 0.001)
SEVERITY	0.335	0.544	0.325	0.540
	(*P* < 0.001)	(*P* < 0.001)	(*P* < 0.001)	(*P* < 0.001)
BENEFITS	0.523		0.523	
	(*P* < 0.001)		(*P* < 0.001)	
BARRIERS		−1.349		−1.354
		(*P* < 0.001)		(*P* < 0.001)
AVAILABILTY	−0.021	−0.015	−0.013	−0.015
	(0.771)	(0.836)	(0.852)	(0.833)
**Information source**				
IS_TRADITIONALMEDIA	−0.251	−0.178	−0.203	−0.166
	(0.006)	(0.046)	(0.033)	(0.073)
IS_SPECIALIZEDHEALTH	0.015	−0.050	0.031	−0.048
	(0.909)	(0.739)	(0.819)	(0.751)
IS_SOCIALMEDIA	−0.167	−0.075		
	(0.013)	(0.299)		
**Social media use for health information**				
SM_SHORTVIDEO			−0.056	−0.010
			(0.361)	(0.885)
SM_COMMUNITY			−0.061	−0.043
			(0.367)	(0.588)
SM_INFORMATION			0.010	−0.004
			(0.895)	(0.964)
SM_ENTERTAINMENT			−0.165	−0.050
			(0.061)	(0.586)
**Governmental efforts**			
GOV_STRATEGY	−0.245	−0.227	−0.271	−0.236
	(0.028)	(0.103)	(0.017)	(0.085)
GOV_PERFORMANCE	−0.198	−0.085	−0.185	−0.084
	(0.004)	(0.231)	(0.010)	(0.244)
**Knowledge of COVID-19**				
KNOW_SYMPTOMS	−0.043	−0.032	−0.031	−0.025
	(0.446)	(0.582)	(0.589)	(0.670)
KNOW_TREATMENTS	0.137	0.236	0.126	0.231
	(0.023)	(0.001)	(0.040)	(0.002)
**Demographic characteristics**				
Female	0.218	0.151	0.229	0.156
	(0.115)	(0.302)	(0.098)	(0.283)
28–37 years	0.087	0.176	0.125	0.193
	(0.552)	(0.269)	(0.403)	(0.236)
38–47 years	0.473	0.481	0.565	0.526
	(0.030)	(0.029)	(0.008)	(0.018)
48–57 years	0.799	0.372	0.904	0.421
	(0.006)	(0.216)	(0.001)	(0.157)
>58 years old	0.268	−0.132	0.413	−0.086
	(0.364)	(0.691)	(0.204)	(0.807)
Black or Latino	−0.385	−0.357	−0.366	−0.347
	(0.204)	(0.203)	(0.222)	(0.220)
Asian	0.715	1.270	0.759	1.282
	(0.012)	(*P* < 0.001)	(0.009)	(*P* < 0.001)
Middle Eastern	−0.326	−0.021	−0.340	−0.022
	(0.170)	(0.934)	(0.154)	(0.931)
Between $30,000 and 50,000	0.100	0.108	0.087	0.101
	(0.630)	(0.629)	(0.678)	(0.649)
Between $50,000 and 80,000	−0.130	−0.272	−0.147	−0.286
	(0.545)	(0.222)	(0.500)	(0.198)
Between $80,000 and 100,000	−0.302	0.032	−0.296	0.030
	(0.140)	(0.895)	(0.157)	(0.903)
Between $100,000 and 150,000	−0.010	0.216	0.034	0.284
	(0.966)	(0.322)	(0.886)	(0.190)
Higher than $150,000	0.087	0.251	0.085	0.243
	(0.651)	(0.206)	(0.655)	(0.219)
Europe	0.415	0.308	0.461	0.320
	(0.308)	(0.504)	(0.251)	(0.482)
Middle East	0.542	0.083	0.511	0.067
	(0.070)	(0.782)	(0.087)	(0.822)
North America	0.809	0.591	0.810	0.598
	(0.017)	(0.105)	(0.019)	(0.098)
cut1	−0.952	−2.652	−0.835	−2.619
	(0.102)	(*P* < 0.001)	(0.158)	(*P* < 0.001)
cut2	−0.050	−1.670	0.056	−1.643
	(0.932)	(0.007)	(0.923)	(0.007)
cut3	0.916	−0.509	1.019	−0.482
	(0.111)	(0.406)	(0.079)	(0.426)
cut4	1.624	0.408	1.728	0.437
	(0.005)	(0.508)	(0.003)	(0.473)
Observations	372	372	372	372
Pseudo-*R*^2^	0.221	0.326	0.219	0.325
Wald Chi Square (30)	225.597	311.698	239.368	314.968
Prob > Chi-sq.	0.000	0.000	0.000	0.000

a *P-values in parentheses*.

**Figure 2:**
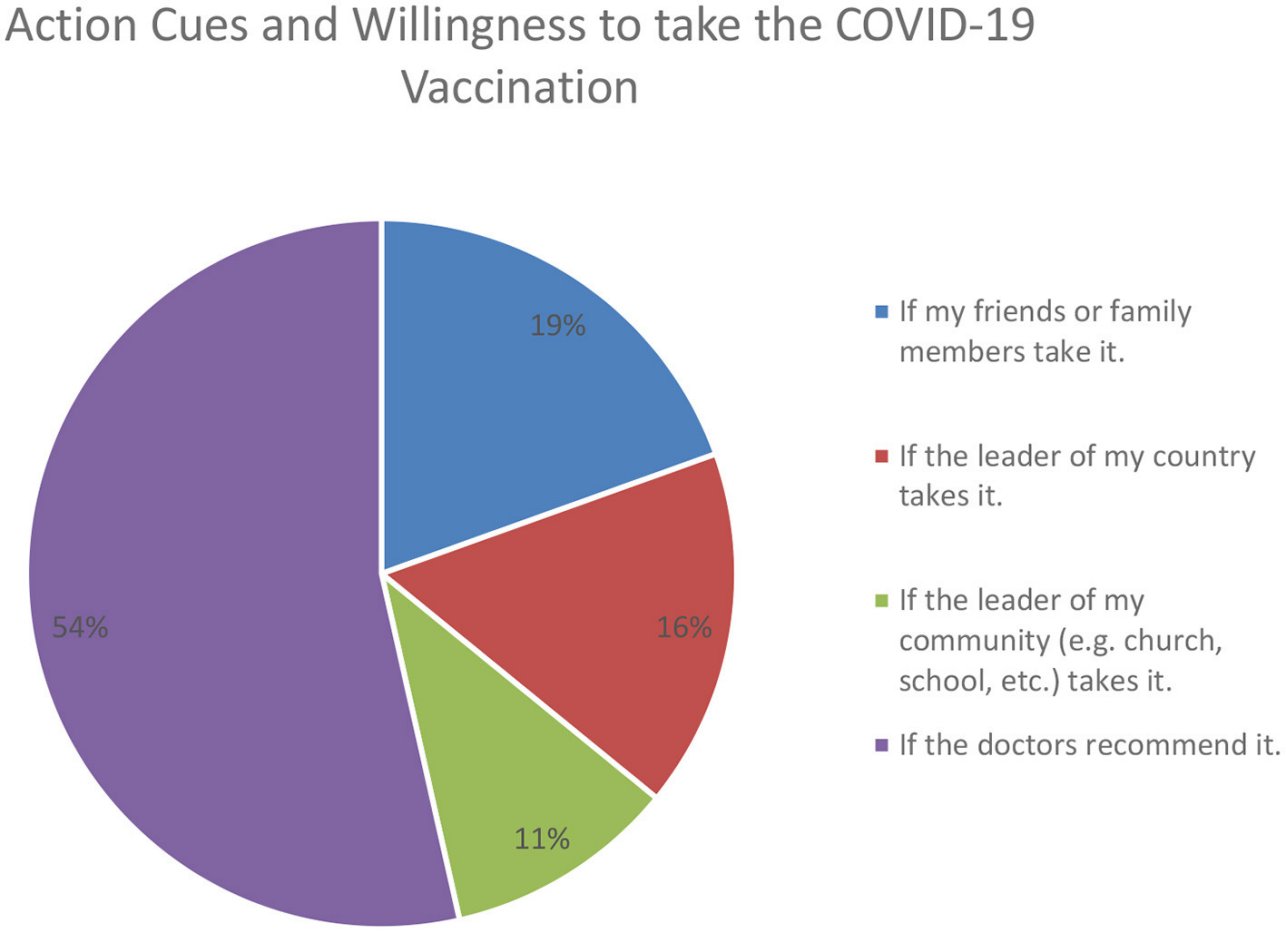
Willingness to vaccinate and action cues from others.

The first set of findings are relevant to the HBM model. ACTION CUES was positively associated with INTENT (*P* < 0.001) on all four models. This finding suggests that individuals are more willing to vaccinate when they see other leaders in their community first getting vaccinated. Citizens' intent to vaccinate is positively impacted by the action of other opinion leaders in the community. For example, Figure 2 displays the total willingness to vaccinate if: (1) friends or family take it, (2) leader of the country takes it (3) leader of the community takes it, and (4) doctors recommend it. Figure 2 shows that doctors seem to have the highest impact as action cues in vaccine intent, followed by friends or family members, leader of the country, and lastly, leader of the community. Next, SEVERITY was also positively associated with INTENT (*P* < 0.001) on all four models, displaying that the higher the perceived severity of contracting the COVID-19 disease, the higher the willingness to vaccinate. BENEFITS of the vaccination also positively influenced the willingness to vaccinate (*P* < 0.001), indicating the importance of communicating the benefits of vaccination. Lastly, BARRIERS was negatively related to INTENT (*P* < 0.001). This finding displays that individuals are concerned about the safety and side effects of the vaccination, impacting their willingness to vaccinate.

In terms of information source findings, the results indicate that both traditional media and social media as information sources negatively influence individuals' willingness to vaccinate (*P* = 0.006 and *P* = 0.013, respectively). In terms of exploring the impact of the type of social media platform (Table 3 column 3 and 4), as compared to other social media platforms (such as informational, community-based, and short video-based) that had no significant influences on INTENT, social media platforms for entertainment and social purposes (e.g., Whatsapp, Instagram, and YouTube) negatively influences willingness to vaccinate (*P* = 0.061). This finding highlights the types of social media platforms that possibly allow the virality of misinformation regarding the COVID-19 vaccination.

Next, we find the coefficients for governmental efforts in GOV_STRATEGY and GOV_PERFORMANCE as both negative and significant concerning INTENT (*P* = 0.028 and *P* = 0.024, respectively). The public seem to have an overall negative perception of the effectiveness of government mitigation strategy and performance against COVID-19. This, in turn, has negatively impacted their willingness to vaccinate. This negative perception might be due to the uncertainty surrounding the COVID-19 virus; governmental efforts were often misaligned with media coverage and a mix of misinformation, leading to a lack of trust in governmental efforts (78, 82, 83). These findings display that perceived governmental efforts directly impact willingness to vaccinate (84, 85). This finding reflects upon the pandemic's political ramifications (84, 86, 87) that have been introduced and its negative impact on citizens' willingness to vaccinate.

Furthermore, the findings display that there was no significant impact on the knowledge of symptoms of COVID-19. However, there was a significant positive influence of knowledge on the treatment of COVID-19 (KNOW_TREATMENT *P* = 0.023). This finding highlights the importance of knowledge that should be communicated to citizens to enhance their willingness to vaccinate.

Lastly, some demographic controls merit attention in terms of willingness to vaccinate. Age groups 38–47 and 48–57 are more willing to vaccinate than the other age groups (*P* = 0.030 and *P* = 0.006, respectively). Asian ethnicity, as compared to other races, are more willing to vaccinate (*P* = 0.012). Lastly, North American citizens are more willing to vaccinate than citizens of other countries in the sample (*P* = 0.017).

## Discussion

This study finds that, overall, the HBM constructs (action cues, severity, barriers, and benefits), government efforts, sources of information, social media usage, and knowledge of COVID-19 all influence willingness to vaccinate. These are all critical factors needed to reach herd immunity. Disentangling some of these may provide some actionable points to increase herd immunity.

The action cue finding inherently highlights the “wait and see” action cue from others in the community, especially from doctors, followed by friends and family, country leaders, and community leaders. This finding is in line with psychologists who have discussed that individuals may fear the side effects or long-term effects of the vaccination and prefer to “wait and see” (88). People lean toward conformity bias, a desire to agree with others and trust others' judgment (89, 90). Therefore, if the network holds strong anti-vaccine views, then the surrounding network will all be impacted. Thus, it is essential to target specific “heads” of the community to allow for faster dissemination of “willingness” to vaccinate. This finding shed light on how herd immunity can be achieved by initiating vaccinations with certain community heads (e.g., doctors, presidents). To illustrate, even an anti-vax may be supporting a leader in other contexts, who may influence his or her vaccination. Such initiation will allow vaccine-hesitant individuals to ease into higher willingness to vaccinate. This finding highlights the importance of mounting a well-planned vaccination campaign that involves and demonstrates that leaders, doctors, and celebrities support the vaccination. In addition, doctors, scientists, and politicians should also publicly speak in support of the COVID-19 vaccine science.

The finding that perceived severity influences willingness to vaccinate is also crucial to herd immunity. With the mass amount of misinformation on the pandemic, the disease's severity may be undermined, and some people may believe that the vaccine is not needed (49). This finding displays that proper clear messages should be communicated on the severity of the COVID-19 virus and its cause to individuals infected with the disease. This, in turn, will allow for herd immunity to be reached by influencing citizens' willingness to vaccinate.

Furthermore, the benefits of the vaccination should also be well-communicated. The finding highlights that the higher the perceived benefits, the greater the influence on one's intent to vaccinate. This finding is in line with previous studies that display the importance of displaying the vaccination benefits (53, 56, 91). Clarifying the vaccination benefits to the public is one way to displace the misinformation (66, 68). Health authorities and policymakers need to clarify any misinformation about the vaccination to the public and control misinformation. The perceived barriers of vaccination also influence the willingness to vaccinate. This finding suggests that different types of information impact users' willingness to vaccinate. Information about the safety and side effects of the vaccine has a strong impact on one's behavior. This important finding displays how policymakers need to provide more informative insights and disseminate clear messages on such topics.

The next set of findings highlights the importance of information sources on willingness to vaccinate. This study finds that media, both traditional and social media, negatively impact one's willingness to vaccinate. Such finding is significant, displaying the negative impact politics and misinformation has had during the pandemic and its impact on citizens' trust (51, 52, 71). Surprisingly, this is consistent across the sample, indicating an overall negative media impact. Social media companies need to police their networks and eliminate false information about the COVID-19 vaccine. Trusted scientists and politicians need to speak in support of COVID-19 vaccine science on all media platforms. Lawmakers should do more to regulate sources of misinformation, just as they have done for other threats to health, such as tobacco (90).

Digging deeper into social media, the study finds that specific social media platforms are used more for entertainment purposes (e.g., Whatsapp, Instagram, and YouTube) than informational purposes (e.g., Twitter), hurt the willingness to vaccinate. This finding displays the types of platforms with the stronger virality of misinformation. Therefore, even stricter measures should be taken on such platforms. In addition, this information needs to be well-communicated that such sources should not be trusted. Policymakers and governments should appropriately utilize social media and information channels by spreading clear information on vaccination and its benefits.

To further the above finding on misinformation and distrust, the study displays that negative perception of governmental efforts harm willingness to vaccinate. The pandemic was an information crisis (92), uncertainty, distrust, and fear were further accentuated by the role played by media platforms and in particular, social media in distributing misinformation, which led citizens to speculate on governmental efforts (78, 83, 93). The pandemic seems to not only rely on, but may change, the extent to which people trust institutions (94). This finding goes in line with the lack of trust in general and the pandemic's political ramifications (22, 95). The public seems to have an overall negative perception of all sorts of media, whether from the government or other sources.

Lastly, the study highlights the importance of awareness and knowledge to COVID-19 (66, 93), types of COVID-19 knowledge, and information–treatment information has more substantial influence than others. Communicating information on the methods of “treatment” of COVID-19 influences the willingness to vaccinate. This is a finding that policymakers and influencers can utilize to avail such information to the public to reach herd immunity.

### Limitations

This study examines factors that influence citizens' willingness to vaccinate for COVID-19 at a point in time. However, the citizen might go back and forth in the decision process, and the knowledge level or source of information or other factors may change over time. This is a limitation of this study, as the data set used is a cross-sectional survey. Furthermore, the questionnaire was on the internet. Therefore, respondents are all users of the internet. The study does not examine non-internet users, which could have differential impacts. Thus, the generalization of the sample to a uniform national culture characteristic is a limitation of this study. Future studies could conduct both internet and non-internet surveys and examine the difference in willingness to vaccinate. Due to various resource limitations during the disease crises and movement restrictions worldwide, the study sample is small limiting the generalizability of the findings. However, we note that even with the small sample, the study has significant implications and insights on the current vaccination behavior of people. Lastly, the cross-sectional design may raise concerns about the predictive value of the study. We note that the results, and any mention of “influence” in the paper, should be taken with the associational value.

## Conclusions

Vaccination is a public intervention that will lead to herd immunity (96, 97). The central part of vaccination uptake is public confidence or trust (98). This study points to important predictors of vaccination intent that can help government authorities design and deliver targeted intervention programs to enhance COVID-19 vaccine uptake. Predictors of vaccine intent include action cues of other leaders taking the vaccine, high perceived benefits of the vaccine, high perceived efficacy of the vaccine, high perceived severity of contracting the COVID-19 disease and lower perceived barriers to receiving the vaccine. With the plethora of misinformation, media seems to have an overall negative impact on vaccine intent, and in particular, entertainment-based social media platforms have a more negative influence on vaccine intent than informational-based social media platforms. Controlling and clarifying information is very important as knowledge of COVID-19 treatment positively influences vaccine intent. Furthermore, the misinformation and distress of citizens with the COVID-19 pandemic is apparent with the negative perceived governmental efforts hurting the vaccine intent, which calls for controlling and clarifying information.

## Data Availability Statement

The raw data supporting the conclusions of this article will be made available by the authors, without undue reservation.

## Ethics Statement

Ethical review and approval was not required for the study on human participants in accordance with the local legislation and institutional requirements. The patients/participants provided their written informed consent to participate in this study.

## Author Contributions

AA-H, JK, and DY designed the study. AA-H and DY designed the survey, carried out the statistical analysis and interpretation, and wrote the manuscript. All authors contributed to the article and approved the submitted version.

## Funding

This study was supported and funded by Kuwait University Research Grant Number IQ02/20. We would like to thank Kuwait University's Research Administration for granting the project and facilitating the research implementation.

## Conflict of Interest

The authors declare that the research was conducted in the absence of any commercial or financial relationships that could be construed as a potential conflict of interest.

## Publisher's Note

All claims expressed in this article are solely those of the authors and do not necessarily represent those of their affiliated organizations, or those of the publisher, the editors and the reviewers. Any product that may be evaluated in this article, or claim that may be made by its manufacturer, is not guaranteed or endorsed by the publisher.
