# Supplementary material for: Does Seeing What Others Do Through Social Media Influence Vaccine Uptake and Help in the Herd Immunity Through Vaccination? A Cross-Sectional Analysis
Source: Front Public Health. 2021 Nov 2;9:715931. doi: 10.3389/fpubh.2021.715931 (PMC8592939; doi:10.3389/fpubh.2021.715931)
Supplement: Supplementary file 1 [file Table_1.DOCX]

**Multimedia Appendix 1**

**Table A1.** Survey questionnaire and coding scheme

| **Variable** | **Description** | **Questionnaire Items***.* | **Refs.** |  |
| --- | --- | --- | --- | --- |
| *Dependent Variable* | | | |  |
| INTENT | Individual’s intention to take vaccine | If a vaccine against COVID-19 is made available, would you take it?  *Scale: 1 – Strongly Unlikely to 5-Strongly Likely* | (Hassani et al., 2014; Macdonell et al., 2013; Milne, Sheeran, & Orbell, 2000; Rahaei, Ghofranipour, & Morowatisharifabad, 2015; Han Xiao et al., 2014; H. Xiao et al., 2016; Yan et al., 2014) |  |
| *Independent Variables* | | | |  |
| ACTION CUES | This is the stimulus needed to trigger the decision-making process to accept the COVID-19 vaccine. These cues can be internal (e.g., chest pains, wheezing, etc.) or external (e.g., advice from others, illness of family member, newspaper article, etc.). | 1. I would feel better about the COVID-19 vaccine if it was taken by many in the public. 2. I would take a vaccine against COVID-19 if the leader of my country takes it. 3. I would take a vaccine against COVID-19 if the leader of my community (e.g. church, school, etc.) takes it. 4. I would take a vaccine against COVID-19 if the doctors recommend it (e.g. Dr. Fauci).   *Cronbach Alpha: 0.64.* | (Becker, 1974; Champion, 2008; Rosenstock, 1974) |  |
| SEVERITY | Perceived severity or degree of harm from engaging in unhealthy behavior; the extent to which one will experience suffer or die from contracting Covid-19. | 1. The complications of COVID-19 are serious. 2. I am scared of getting COVID-19. 3. I will be very sick if I get COVID-19.   *Scale: 1 - Strongly Disagree to 5-Strongly Agree.*  *Cronbach Alpha: 0.71.* | (Becker, 1974; Champion, 2008; Rosenstock, 1974) |  |
| BENEFITS | This refers to a person's perception of the effectiveness of the available vaccination to reduce the threat of COVID-19 disease. | 1. Vaccinations decreases the chance of infections or complications. 2. The COVID-19 vaccine makes me feel less worried.   *Scale: 1 - Strongly Disagree to 5-Strongly Agree Cronbach Alpha: 0.77.* | (Becker, 1974; Champion, 2008; Rosenstock, 1974) |  |
| BARRIERS | This refers to a person's feelings on the obstacles on taking the COVID-19 vaccine. | 1. I am concerned about the safety of the COVID-19 vaccine. 2. I am concerned about the side-effects of the COVID-19 vaccine that might interfere with my daily activities. 3. I will not take the vaccine because of the possible side effects of the vaccine   *Scale: 1 - Strongly Disagree to 5-Strongly Agree Cronbach Alpha: 0.80.* | (Becker, 1974; Champion, 2008; Rosenstock, 1974) |  |
| EFFICACY | Perceived of the vaccine efficacy against Covid-19 | Do you believe that taking the vaccine will protect you from Covid-19 in the future?  *Scale: 1 - Strongly Disagree to 5-Strongly Agree.* | (Hassani et al., 2014; Macdonell et al., 2013; Milne et al., 2000; Rahaei et al., 2015) |  |
| AVAILABILITY | Vaccine availability | The vaccine will be available near me:  *Scale: 1 – Immediately to 5 – Never* | (Hassani et al., 2014; Macdonell et al., 2013; Milne et al., 2000; Rahaei et al., 2015) |  |
| COVID-19  INFORMATION SOURCES | The extent to which an individual uses health information sources to attain COVID-19 information. | Which of the following do you use for Covid-19 related information? Check all that apply (a) Social Media, (b) TV, (c) Newspaper (online and print), (d) Friends or Family, (e) Doctors or Medical Professionals.  *Count of total number of information sources used was used to code the COVID-19 INFORMATION SOURCE variable.* |  |  |
| COVID-19 SOCIAL MEDIA | The extent to which an individual uses social media to attain COVID-19 information. | Which of the following social media do you use for Covid-19 news? Check all that apply (a) Facebook, (b) Twitter, (c) WhatsApp, (d) Instagram, (e) LinkedIn, (f) Snapchat, (g) TikTok, (h) YouTube, (i) Other.  *Count of total number of social media platforms used to attain COVID-19 information was used to code the COVID-19 SOCIAL MEDIA variable.* |  |  |
| KNOWLEDGE-SYMPTOMS | The extent to which one is aware or knowledgeable about COVID-19 and relevant situations. | *Which of the following are symptoms of Covid-19? (a) Fever, (b) Cough, (c) Difficulty Breathing, (d) Nausea, (e) Diarrhea, (f) Skin Rash, (g) Loss of smell and taste*  *The total number of selected choices were added* as KNOWLEDGE – SYMPTOMS variable*.* | ("Corona Virus Disease 2019 (COVID-19) Frequently Asked Questions," 2020) |  |
| GOVERNMENT MITIGATION STRATEGY | Perceived effectiveness of government strategy | *What do you think is the best way to slow down the spread of Covid-19?*  *Scale: 1 – Wearing masks and social distancing, 2 – Quarantining at home, 3 – Shutting down borders, highways, to restrict travel* |  |  |
| GOVERNMENT PERFORMANCE | Perceived effectiveness of government performance against Covid-19 policy | *In your opinion, who is most responsible, if any, for the spread and deaths caused by Covid-19?*  *Scale: 1 – Terrible to 5 – Great job overall* |  |  |
| KNOWLEDGE-TREATMENTS | The extent to which one is aware or knowledgeable about COVID-19 and relevant situations. | *Which of the following are effective treatments of Covid-19? (a) Drink fluid and getting rest, (b) Hydroxychloroquine, (c) Herbal Medicine, (d) Vaccines, (f) Remdisivir, (g) Corticosteroids, (h) Convalexcent Plasma*  *The total number of selected choices were added* *and then* *subtracted by the total number of incorrect choices selected – (b), (g), (h).* | ("Corona Virus Disease 2019 (COVID-19) Frequently Asked Questions," 2020) |  |
| *Control Variables* | | | |  |
| AGE | Age of respondent | My age group is: (a) 18-27 years old, (b) 28-37 years old, (c) 38-47 years old, (d) 48-57 years old, (e) greater than 58 years old  *The variable was coded by taking the responses to the question, where the checked responses to the ordered category choice sets (i.e., five) were transformed into corresponding numerical values.* | |  |
| COUNTRY | Region of residence | I reside in: (a) North America, (b) Middle East, (c) Europe, (d) Asia.  *Dummy variables were coded for countries based on responses.* | |  |
| GENDER | Gender of respondent | I am: (a) Male, (b) Female  *The variable was coded by Female = 1 and Male = 0.* | |  |
| INCOME | Household income of the respondent | My household income is: (a) Less than $30,000, (b) $30,000 - $50,000, (c) $50,000 - $80,000, (d) $80,000 - $100,000, (e) $100,000 - $150,000, (f) Higher than $150,000  *The variable was coded by taking the responses to the question, where the checked responses to the ordered category choice sets (i.e. six) were transformed into corresponding numerical values.* | |  |
| ETHNICITY | Ethnicity of the respondent | I would describe myself as: (a) White or Caucasian, (b) Black of Latinx, (c) Asian, (d) Middle Eastern | |  |

**Table A2.** Summary statistics of key variables (N=378).

| **Variable** | **Frequency** | **Percentage** |
| --- | --- | --- |
| AGE GROUP - 18-27 | 125 | 33.1% |
| AGE GROUP - 28-37 | 145 | 38.4% |
| AGE GROUP - 38-47 | 64 | 16.9% |
| AGE GROUP - 48-57 | 21 | 5.6% |
| AGE GROUP - 58+ | 23 | 6.1% |
| ETHNICITY – MIDDLE EASTERN | 244 | 64.9% |
| ETHNICITY – WHITE OR CAUCASIAN | 84 | 22.3% |
| ETHNICITY – BLACK OR LATINX | 30 | 8.0% |
| ETHNICITY – ASIAN | 18 | 4.8% |
| FEMALE | 224 | 59.9% |
| HOUSEHOLD INCOME - Less than $30K | 81 | 21.4% |
| HOUSEHOLD INCOME - $30K-$50K | 54 | 14.3% |
| HOUSEHOLD INCOME - $50K - $80K | 57 | 15.1% |
| HOUSEHOLD INCOME - $80K-$100K | 47 | 12.4% |
| HOUSEHOLD INCOME - $100K-$150K | 50 | 13.2% |
| HOUSEHOLD INCOME - $150K or more | 89 | 23.5% |
| REGION – ASIA | 14 | 3.7% |
| REGION – EUROPE | 15 | 4.0% |
| REGION – NORTH AMERICA | 238 | 63.0% |
| REGION – MIDDLE EAST | 111 | 29.4% |

**Table A3.** Interitem correlation and Cronbach’s alpha.

| Item  ACTION CUES | Obs. | Sign | item-test correlation | item-rest correlation | average interitem correlation | alpha |
| --- | --- | --- | --- | --- | --- | --- |
| Item 1 | 378 | + | 0.66 | 0.42 | 0.26 | 0.58 |
| Item 2 | 378 | + | 0.69 | 0.47 | 0.24 | 0.56 |
| Item 3 | 378 | + | 0.66 | 0.43 | 0.26 | 0.58 |
| Item 4 | 378 | + | 0.63 | 0.39 | 0.27 | 0.60 |
| **Test scale** |  |  |  |  | **0.27** | **0.64** |
| Item  SEVERITY | Obs. | Sign | item-test correlation | item-rest correlation | average interitem correlation | alpha |
| Item 1 | 378 | + | 0.74 | 0.43 | 0.60 | 0.75 |
| Item 2 | 378 | + | 0.84 | 0.61 | 0.36 | 0.53 |
| Item 3 | 378 | + | 0.81 | 0.56 | 0.41 | 0.58 |
| **Test scale** |  |  |  |  | **0.45** | **0.71** |
| Item  BENEFITS | Obs. | Sign | item-test correlation | item-rest correlation | average interitem correlation | alpha |
| Item 1 | 378 | + | N/A | N/A | N/A | N/A |
| Item 2 | 378 | + | N/A | N/A | N/A | N/A |
| **Test scale** |  |  |  |  | **0.63** | **0.77** |
| Item  BARRIERS | Obs. | Sign | item-test correlation | item-rest correlation | average interitem correlation | alpha |
| Item 1 | 378 | + | 0.86 | 0.68 | 0.54 | 0.70 |
| Item 2 | 378 | + | 0.88 | 0.72 | 0.48 | 0.65 |
| Item 3 | 378 | + | 0.79 | 0.55 | 0.71 | 0.83 |
| **Test scale** |  |  |  |  | **0.57** | **0.80** |

**Table A4.** Factor Analysis –Health Information Source

| (Obs.: 378, Principal Factors, Retained: 3, Rotation: None, # of Param.: 10) | | | | |
| --- | --- | --- | --- | --- |
| Factor | Eigenvalue | Difference | Proportion | Cumulative |
| Factor1 | 0.551 | 0.250 | 1.254 | 1.254 |
| Factor2 | 0.301 | 0.279 | 0.685 | 1.938 |
| Factor3 | 0.022 | 0.188 | 0.050 | 1.988 |
| Factor4 | -0.166 | 0.103 | -0.378 | 1.611 |
| Factor5 | -0.268 | . | -0.611 | 1.000 |
| LR test: chi2(10) | 119.65 |  | Prob>chi2 | 0 |

**Table A5.** Factor Loadings –Health Information Source

| Health Information Source | | | | |
| --- | --- | --- | --- | --- |
| Variable | Factor1 | Factor2 | Factor3 | Uniqueness |
| Social Media | 0.216 | -0.401 | -0.004 | 0.793 |
| TV | 0.222 | 0.196 | 0.108 | 0.901 |
| Newspaper | 0.386 | 0.205 | -0.012 | 0.809 |
| Friends/Family | 0.493 | -0.165 | 0.014 | 0.730 |
| Doctors | 0.251 | 0.180 | -0.100 | 0.894 |

**Table A6.** Factor Analysis – Social Media Use for COVID-19 Information

| (Obs.: 378, Principal Factors, Retained: 4, Rotation: None, # of Param.: 26) | | | | |
| --- | --- | --- | --- | --- |
| Factor | Eigenvalue | Difference | Proportion | Cumulative |
| Factor1 | 1.094 | 0.707 | 1.171 | 1.171 |
| Factor2 | 0.387 | 0.318 | 0.414 | 1.585 |
| Factor3 | 0.069 | 0.054 | 0.074 | 1.659 |
| Factor4 | 0.015 | 0.061 | 0.016 | 1.675 |
| Factor5 | -0.046 | 0.081 | -0.050 | 1.625 |
| Factor6 | -0.127 | 0.071 | -0.136 | 1.489 |
| Factor7 | -0.199 | 0.060 | -0.212 | 1.277 |
| Factor8 | -0.258 | . | -0.277 | 1.000 |
| LR test: chi2(28) | 209.49 |  | Prob>chi2 | 0 |

**Table A7.** Factor Loadings – Social Media Use for COVID-19 Information

| Social Media Use for COVID-19 Information | | | | |
| --- | --- | --- | --- | --- |
| Variable | Factor1 | Factor2 | Factor3 | Uniqueness |
| Facebook | 0.222 | 0.358 | 0.064 | 0.012 |
| Twitter | 0.105 | -0.293 | 0.012 | 0.058 |
| Instagram | 0.494 | -0.159 | 0.025 | 0.044 |
| WhatsApp | 0.528 | -0.098 | 0.039 | -0.042 |
| LinkedIn | 0.190 | 0.308 | 0.093 | 0.051 |
| Snapchat | 0.536 | -0.010 | -0.112 | 0.018 |
| TikTok | 0.315 | 0.183 | -0.150 | -0.031 |
| YouTube | 0.299 | -0.099 | 0.138 | -0.060 |

Becker, M. (1974). The Health Belief Model and personal health behavior. . *2*, 324–508.

Champion, V., Skinner, CS. . (2008). *The Health Belief Model* (Vol. 4). San Francisco, CA: Jossey-Bass.

Corona Virus Disease 2019 (COVID-19) Frequently Asked Questions. (2020). Retrieved from <https://www.cdc.gov/coronavirus/2019-ncov/faq.html?CDC_AA_refVal=https%3A%2F%2Fwww.cdc.gov%2Fcoronavirus%2F2019-ncov%2Fprepare%2Fchildren-faq.html>

Hassani, L., Dehdari, T., Hajizadeh, E., Shojaeizadeh, D., Abedini, M., & Nedjat, S. (2014). Development of an instrument based on the protection motivation theory to measure factors influencing women's intention to first pap test practice. *Asian Pac J Cancer Prev, 15*(3), 1227-1232. doi:10.7314/apjcp.2014.15.3.1227

Macdonell, K., Chen, X., Yan, Y., Li, F., Gong, J., Sun, H., . . . Stanton, B. (2013). A Protection Motivation Theory-Based Scale for Tobacco Research among Chinese Youth. *J Addict Res Ther, 4*, 154. doi:10.4172/2155-6105.1000154

Milne, S., Sheeran, P., & Orbell, S. (2000). Prediction and intervention in health‐related behavior: A meta‐analytic review of protection motivation theory. *Journal of applied social psychology, 30*(1), 106-143. doi:10.1111/j.1559-1816.2000.tb02308.x

Rahaei, Z., Ghofranipour, F., & Morowatisharifabad, M. A. (2015). Psychometric properties of a protection motivation theory questionnaire used for cancer early detection. *Journal of School of Public Health and Institute of Public Health Research, 12*(3), 69-79.

Rosenstock, I. M. (1974). Historical origins of the health belief model. *Health education monographs, 2*(4), 328-335.

Xiao, H., Li, S., Chen, X., Yu, B., Gao, M., Yan, H., & Okafor, C. N. (2014). Protection motivation theory in predicting intention to engage in protective behaviors against schistosomiasis among middle school students in rural China. *PLoS neglected tropical diseases, 8*(10). doi:10.1371/journal.pntd.0003246

Xiao, H., Peng, M., Yan, H., Gao, M., Li, J., Yu, B., . . . Li, S. (2016). An instrument based on protection motivation theory to predict Chinese adolescents' intention to engage in protective behaviors against schistosomiasis. *Glob Health Res Policy, 1*, 15. doi:10.1186/s41256-016-0015-6

Yan, Y., Jacques-Tiura, A. J., Chen, X., Xie, N., Chen, J., Yang, N., . . . Macdonell, K. K. (2014). Application of the protection motivation theory in predicting cigarette smoking among adolescents in China. *Addict Behav, 39*(1), 181-188. doi:10.1016/j.addbeh.2013.09.027
